# Ferrichrome: bacterial function, synthetic mimics, and biomedical applications

**DOI:** 10.3389/fmicb.2026.1932981

**Published:** 2026-09-23

**Authors:** Nicolas Räther, Monika Wojciechowska, Joanna Trylska, Nadja Patenge

**Affiliations:** 1 Institute for Medical Microbiology, Virology and Hygiene, University Medicine Rostock, Rostock, Germany; 2 Centre of New Technologies, University of Warsaw, Warsaw, Poland

**Keywords:** antimicrobials, ferrichrome, ferrichrome analogs, ferrichrome synthesis, ferrichrome uptake in bacteria, iron chelator, medical imaging, siderophore

## Abstract

Iron is an essential micronutrient for most living organisms, but its bioavailability is limited. To facilitate iron uptake, many bacteria and fungi secrete siderophores, which are small, high-affinity iron-chelating molecules. Siderophores play a key role in microbial competition and immune evasion, and additionally serve as an entry point for bacteriophages and toxins. Ferrichrome, a well-studied fungal hydroxamate siderophore, is utilized as an iron source by numerous bacterial species, including human-associated and pathogenic bacteria. Ferrichrome is a cyclic hexapeptide composed of three glycine residues and three N^5^-acetyl-N^5^-hydroxy-L-ornithine residues, which provide hydroxamate groups that are responsible for iron chelation. Synthetic strategies for desferrichrome and hydroxamate siderophore scaffolds were developed over time allowing the utilization of orthogonally protected hydroxyornithine building blocks. Modern orthogonal solid-phase peptide synthesis supports structural investigation and allows precise functionalization for the design of ferrichrome analogs. Inspired by naturally occurring siderophore-antibiotic conjugates that exploit siderophore uptake pathways to enter bacterial cells, synthetic ferrichrome or ferrichrome mimics have been coupled to a variety of antibacterial moieties. Ferrichrome conjugation to antibiotics improved bacterial uptake and achieved MICs in the nanomolar range. Ga-complexed ferrichrome mimics did not perturb expression of siderophore-transport genes and showed even higher antibacterial activity. In a proof-of-concept study, antisense oligonucleotides coupled to ferrichrome analogs were transported into *E. coli* and gene silencing could be monitored by a decrease in fluorescence. Ferrichrome has also been employed as a metal chelator in preclinical zirconium- and gallium-based PET imaging agents. ^68^Ga-ferrirubin demonstrated favorable properties for imaging bacterial infection caused by *S. aureus* and *A. baumannii*. Ferrichrome has exhibited antitumor activity against different tumor entities *in vitro* and in murine xenograft models. The mechanistic background of the antitumor effects is not yet comprehensively understood and the investigation of immune effects is hampered by the restricted choice of suitable *in vivo* models. Overall, ferrichrome and its mimics are versatile molecules with strong potential for analytical and therapeutic applications, but further preclinical studies are required in the respective fields to establish the suitability of the molecules.

## 1 Introduction

Iron is essential for the growth of nearly all microbial lifeforms. In bacteria, it is involved in many biological processes, such as DNA replication, amino acid biosynthesis, and the tricarboxylic acid cycle (Vogt et al., 2021). Iron predominantly occurs in the insoluble ferric form, thereby limiting iron bioavailability. In the human body, access is further restricted because most iron is either bound intracellularly within hemoglobin or to proteins such as transferrin, lactoferrin or albumin (Aisen et al., 1978; Vogt et al., 2021). During infection, iron is even more efficiently sequestered as part of the innate immune response. Human pathogenic bacteria have evolved diverse strategies to acquire iron, including heme acquisition systems, transferrin receptors, and the secretion of siderophores, small, high-affinity Fe(III)-binding compounds (50-−1500 Da) (Ullah and Lang, 2023).

Bacteria, filamentous fungi, yeasts, and several plants secrete siderophores in response to iron deprivation. Siderophores capture ferric iron, compete with other chelating compounds in the environment, and are taken up via specialized active transporters. Bacterial siderophores have been comprehensively reviewed elsewhere (Gräff and Barry, 2024; Kumar et al., 2025; Schalk, 2025). Ferrichrome, initially isolated from the smut fungus *Ustilago sphaerogena* (Neilands, 1952), was one of the first siderophores identified and represents a prototypical hydroxamate-type siderophore.

Based on their iron-chelating moieties, siderophores are commonly categorized, although not always consistently, into hydroxamates, catecholates, carboxylates, phenolates, and mixed-type siderophores (Timofeeva et al., 2022). Typically, siderophores consist of three bidentate groups and employ negatively charged oxygen atoms to bind ferric iron, forming a stable octahedral geometry around the iron center (Hider and Kong, 2010). Ferrichrome is a cyclic hexapeptide trihydroxamate siderophore composed of three glycine residues and three N^5^-acetyl-N^5^-hydroxy-L-ornithine residues that coordinate Fe(III) through six oxygen atoms in an octahedral geometry (van der Helm et al., 1980; Constantine et al., 1990). The core structure of ferrichrome is used as a reference for classifying several structurally related compounds as ferrichrome-type siderophores, collectively forming the ferrichrome family. While the designation “ferrichrome family” was originally introduced by Neilands in 1981, terms such as “ferrichrome-type,” “ferrichrome class,” and “ferrichromes” are still used interchangeably, highlighting the need for a more consistent terminology (Neilands, 1981; Chiu et al., 2022; Happacher et al., 2023). The ferrichrome family includes more than 20 different compounds, representing one of the largest hydroxamate siderophore families. It features a chiral cyclic hexapeptide core asymmetrically substituted with three chelating arms. Amino acid replacements in the ring, and variations in acylation of the ornithine arms are the primary ways in which ferrichrome–type siderophores diverge from ferrichrome (Renshaw et al., 2002; Winkelmann, 2007; Szebesczyk et al., 2016).

Ferrichrome-type siderophores are one of the most frequently produced hydroxamate species by soil-inhabiting fungi, with concentrations in the soil in the nanomolar range (Ahmed and Holmström, 2014; Olofsson and Bylund, 2015). Their cyclic hexapeptide scaffold is expected to confer increased stability against environmental degradation, which may contribute to their persistence in soil and biomedical potential, although direct studies of their environmental stability are lacking (Kaltashov et al., 1997; Winkelmann, 2007). A recent study investigated ferrichrome degradation in marine-derived bacterial cultures but observed iron removal rather than degradation of the ferrichrome scaffold (Monge-Loría et al., 2025). Ferrichromes and other fungal siderophores have been identified in microbially processed cheese, suggesting that fungal siderophores are also present in other fermented foods (Ong and Neilands, 1979; Emri et al., 2013).

Ferrichromes and ferrichrome analogs have been extensively studied in different areas of medical research because of their ability to chelate metals and their potential use as drug carriers. Recent progress included applications ranging from novel antibiotics to anticancer and imaging agents. This review aims to provide an overview of ferrichrome, highlighting its biological role in bacteria, the challenges faced in developing analogs, and relevant areas in medical research.

## 2 Biological role of ferrichrome in bacteria

### 2.1 Ferrichrome uptake by bacteria

Siderophores are taken up by bacteria through active transport across the membrane. Although the specific transport systems vary, the underlying mechanisms are similar across different siderophore types. In Gram-negative bacteria, siderophore uptake is mediated by outer membrane TonB-dependent transporters (TBDTs), which obtain energy from the proton motive force via the TonB–ExbB–ExbD complex and transport siderophores into the periplasm. The TBDTs exhibit high specificity for certain siderophores with additional strain-to-strain variability in siderophore recognition (Ratliff et al., 2022; Schalk, 2025). From the periplasm, siderophores are transported to the cytoplasm through specific ABC transporters (Delepelaire, 2019) (Figure 1A). In Gram-positive bacteria, uptake involves cytoplasmic membrane-anchored receptors that bind siderophore–iron complexes and transfer them to ABC transporters, with specificity comparable to that of Gram-negative systems (Sheldon and Heinrichs, 2015) (Figure 1B). The mechanism of siderophore transport in Gram-positive bacteria remains less well characterized, although an iron shuttle model has been proposed (Fukushima et al., 2014; Endicott et al., 2020).

**Figure 1:**
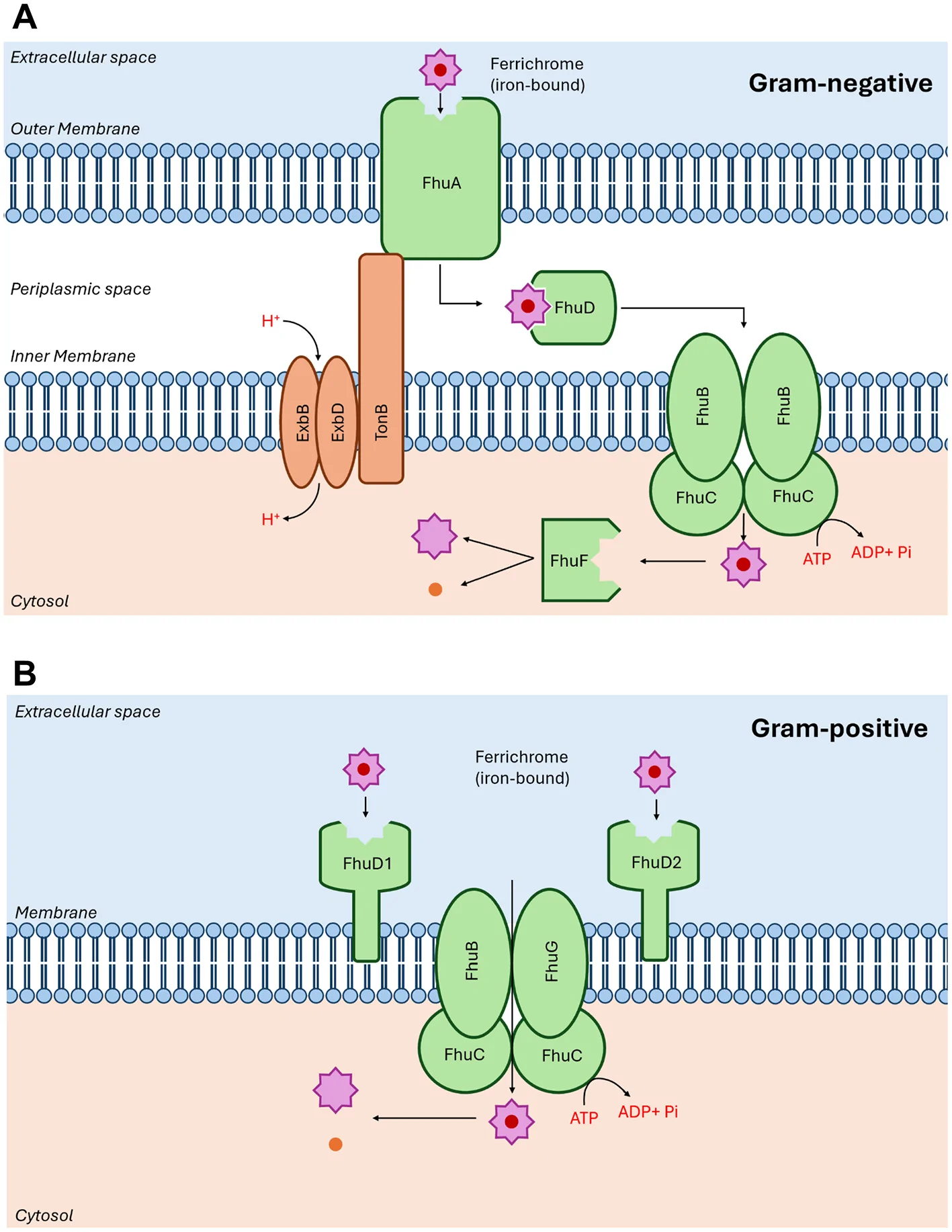
Schematic overview of bacterial ferrichrome uptake. **(A)** Uptake of iron-bound ferrichrome in Gram-negative bacteria, shown for *E. coli*. Iron-bound ferrichrome is recognized and transported across the outer membrane by TonB-dependent transporter (TBDT) FhuA. Energy is provided by the TonB-ExbB-ExbD complex (Eisenhauer et al., 2005; Celia et al., 2020). The periplasmic binding protein (PBP) FhuD captures ferrichrome and delivers it to the inner membrane ATP-binding cassette (ABC) transporter FhuBC, which translocates the siderophore into the cytosol. Iron is released from ferrichrome via enzymatic reduction by FhuF (Braun, 2003; Hu and Zheng, 2021). **(B)** Uptake of iron-bound ferrichrome in Gram-positive bacteria, exemplified by *S. aureus*. Iron-bound ferrichrome in the extracellular space is recognized and bound by the membrane-anchored siderophore-binding proteins FhuD1 or FhuD2 (Sebulsky et al., 2000, 2003) and transported into the cytoplasm through a complex of the heterodimeric permease FhuBG and the ATPase FhuC (Hammer and Skaar, 2011; Ghssein and Ezzeddine, 2022). Small orange circle: ferric iron (Fe^3+^), small red circle: ferrous iron (Fe^2+^), light green: proteins directly involved in ferrichrome transport, light orange: TonB–ExbB–ExbD complex.

The ferric hydroxamate uptake (Fhu) system is responsible for transporting hydroxamate siderophores, including ferrichrome, and is conserved across multiple bacterial species. In Gram-negative bacteria, such as *Escherichia coli*, FhuA acts as the outer membrane receptor recognizing and transporting ferrichrome into the periplasm, using the energy from the TonB-ExbB-ExbD complex. Subsequently, FhuD shuttles ferrichrome and other hydroxamate siderophores to the ABC transporter FhuBC for cytoplasmic translocation (Braun, 2003; Carter et al., 2006; Hu and Zheng, 2021). In Gram-positive bacteria, such as *Bacillus subtilis* and *Staphylococcus aureus*, the siderophore-binding protein FhuD is anchored in the membrane. It binds iron-loaded siderophores, which are then translocated into the cytoplasm by the heterodimeric permease FhuBG and the ATPase FhuC (Schneider and Hantke, 1993; Cabrera et al., 2001). The uptake of ferrichrome has been experimentally demonstrated in numerous bacterial species, as summarized in Table 1.

**Table 1:** List of bacterial species and their ferrichrome uptake systems identified based on experimental studies.

Species	Phylum/Class	Gram classification	Ferrichrome uptake system	References
*Actinobacillus pleuropneumoniae*	Pseudomonadota/ Gammaproteobacteria	Gram-negative	FhuACDB	Diarra et al., 1996; Mikael et al., 2002, 2003
*Actinobacillus suis*	Pseudomonadota/ Gammaproteobacteria	Gram-negative	n. i.	Mikael et al., 2003
*Bacillus cereus*	Bacillota/Bacilli	Gram-positive	FhuBGCD, YxeB	Zawadzka et al., 2009
*Bacillus halodurans*	Bacillota/Bacilli	Gram-positive	n. i.	Miethke et al., 2011
*Bacillus subtilis*	Bacillota/Bacilli	Gram-positive	FhuBGCD	Schneider and Hantke, 1993; Miethke et al., 2011
*Bacteroides fragilis*	Bacteroidota/Bacteroidia	Gram-negative	Fch1, Fch2, FeoAB, XusAB	Rocha and Krykunivsky, 2017; Silale et al., 2026
*Bordetella bronchiseptica*	Pseudomonadota/ Gammaproteobacteria	Gram-negative	n. i.	Beall and Hoenes, 1997
*Bordetella pertussis*	Pseudomonadota/ Gammaproteobacteria	Gram-negative	FbpA, FbpBC	Beall and Hoenes, 1997; Brickman et al., 2011; Banerjee et al., 2014
*Burkholderia cenocepacia*	Pseudomonadota/ Betaproteobacteria	Gram-negative	FeuA, FhuA, FhuB	Hussein et al., 2024
*Clostridoides difficile*	Bacillota/Clostridia	Gram-positive	FhuDBGC	Hastie et al., 2023
*Escherichia coli*	Pseudomonadota/ Gammaproteobacteria	Gram-negative	FhuACDB	Wayne and Neilands, 1975; Hartmann and Braun, 1980
*Haemophilus parasuis*	Pseudomonadota/ Gammaproteobacteria	Gram-negative	FhuABCD	Mikael et al., 2003; del Río et al., 2006
*Listeria monocytogenes*	Bacillota/Bacilli	Gram-positive	FhuBCDG	Jin et al., 2006; Klebba et al., 2012
*Mycobacterium fortuitum*	Actinomycetota/ Actinomycetia	Gram-positive	n. i.	Matzanke et al., 1997
*Mycobacterium smegmatis*	Actinomycetota /Actinomycetia	Gram-positive	n. i.	Matzanke et al., 1997
*Nontypable Haemophilus influenzae*	Pseudomonadota/ Gammaproteobacteria	Gram-negative	FhuABCD	Morton et al., 2010
*Pantoea agglomerans*	Pseudomonadota/ Gammaproteobacteria	Gram-negative	n. i.	Berner et al., 1988
*Pseudomonas aeruginosa*	Pseudomonadota/ Gammaproteobacteria	Gram-negative	FiuABC, FpvB	Llamas et al., 2006; Hannauer et al., 2010; Chan and Burrows, 2023
*Pseudomonas putida*	Pseudomonadota/ Gammaproteobacteria	Gram-negative	n. i.	Jurkevitch et al., 1992
*Salmonella Paratyphi*	Pseudomonadota/ Gammaproteobacteria	Gram-negative	FhuA	Killmann et al., 2001
*Salmonella Typhimurium*	Pseudomonadota/ Gammaproteobacteria	Gram-negative	FhuACDB	Luckey et al., 1972; Tsolis et al., 1995
*Serratia marcescens*	Pseudomonadota/ Gammaproteobacteria	Gram-negative	n. i.	Gaisser and Braun, 1991
*Staphylococcus aureus*	Bacillota/Bacilli	Gram-positive	FhuACDB, FhuD1, FhuD2	Sebulsky et al., 2000, 2003; Cabrera et al., 2001; Sebulsky and Heinrichs, 2001
*Streptococcus pneumoniae*	Bacillota/Bacilli	Gram-positive	PiaABCD^*^	Brown et al., 2002; Pramanik and Braun, 2006; Cheng et al., 2013
*Streptococcus pyogenes*	Bacillota/Bacilli	Gram-positive	FtsABCD	Hanks et al., 2005
*Streptomyces ambofaciens*	Actinomycetota/ Actinomycetia	Gram-positive	n. i.	Barona-Gómez et al., 2006
*Streptomyces coelicolor*	Actinomycetota/ Actinomycetia	Gram-positive	DesE	Barona-Gómez et al., 2006; Arias et al., 2015
*Yersinia pestis*	Pseudomonadota/ Gammaproteobacteria	Gram-negative	FhuCDB, FcuA	Forman et al., 2007

n. i., not identified (ferrichrome utilization has been demonstrated, but no specific uptake system has been identified).

^*^also referred to as PiuABCD and FhuCDBG.

Upon uptake, iron is reduced and released from siderophores via different strategies, such as enzymatic modification or cleavage (Schalk and Guillon, 2013). Ferric iron reductases have been identified in several bacteria, fungi, and yeasts (Cain and Smith, 2021). In *E. coli*, the ferric iron reductase FhuF releases iron from hydroxamate siderophores, including ferrichrome (Trindade et al., 2021).

### 2.2 Ferrichrome supports immune evasion

Iron plays an important role during infection of humans by bacteria and siderophores have been associated with virulence (Khasheii et al., 2021). Bacterial siderophores are mainly responsible for iron acquisition but can also be involved in tolerance to metal-induced toxicity, reactive oxygen species-induced stress tolerance, and cell signaling (Arnold, 2024).

In competitive microbial environments, social dynamics have emerged around siderophore production and uptake. Frequently, microorganisms utilize siderophores produced by other species, known as xenosiderophores. Xenosiderophore-mediated iron uptake provides an advantage by reducing the cost of siderophore production (Kramer et al., 2020; Kumar et al., 2025). Ferrichrome, which is known to be synthesized by fungi and has been reported in *Lactobacilli*, is widely utilized as a xenosiderophore by bacteria. Current evidence suggests that ferrichrome uptake contributes to immune evasion in humans and simultaneously serves as an entry route for toxins and bacteriophages.

In response to bacterial infection and secretion of siderophores, humans induce a hypoferremic environment and secrete Lipocalin 2 (LCN2), which is capable of sequestering siderophores, thereby impairing the bacterial siderophore-mediated iron acquisition. LCN2 contributes to the host defense in the respiratory tract during *E. coli* infection. Intratracheal challenge of LCN2-deficient mice with *E. coli* resulted in increased mortality and significantly higher pulmonary bacterial burdens compared with wild-type mice (Wu et al., 2010). It also functions as a colonization barrier in the urinary tract mucosa. Following infection with *E. coli* H9049, LCN2-deficient mice exhibited a higher bacterial burden in the bladder compared with wild-type mice (Steigedal et al., 2014). Urinary LCN2 deficiency in humans has been associated with recurring urinary tract infections (Forster et al., 2017). Furthermore, LCN2 is an important component of the mucosal immune defense during intestinal infection with *E. coli*. The expression of LCN2 was increased in a piglet model upon infection with *E. coli* K88 and induction of LCN2 expression or addition of recombinant protein led to growth inhibition in piglet epithelial cells (Guo et al., 2017). It was also identified as a critical factor in the immune response to *Acinetobacter baumannii*. It inhibited *A. baumannii* iron-dependent growth and LCN2-deficient mice were not able to control *A. baumannii*-induced *pneumonia* (Sheldon et al., 2022). Beyond siderophore sequestration, LCN2 is also involved in the innate immune response by supporting neutrophil activity and promoting macrophage migration (Wang et al., 2019).

Siderophores that are able to evade LCN2 sequestration, such as ferrichrome, are known as *stealth siderophores* and confer an advantage to bacteria colonizing within the host environment (Golonka et al., 2019). In contrast, Lipocalin 1, identified in human saliva and tears, can sequester a broader range of siderophores, including ferrichrome, albeit with reduced affinity compared to LCN2. It has been suggested as a mechanism for neutralizing a broad array of fungal hydroxamate siderophores (Fluckinger et al., 2004).

In the human gut, pathogenic *Salmonella enterica serovar* Typhimurium employs the stealth siderophore salmochelin to gain a competitive advantage over commensal bacteria such as *E. coli* during intestinal inflammation (Raffatellu et al., 2009). In turn, uptake of a greater repertoire of LCN2-resistant siderophores seems to be partially responsible for the efficacy of *E. coli* Nissle as a probiotic against *S*. Typhimurium (Deriu et al., 2013). The immune-evasive function of stealth siderophores might also play a role for gut commensal bacteria such as *Bacteroides. Bacteroides fragilis* can utilize ferrichrome as an iron source (Rocha and Krykunivsky, 2017). *Bacteroides thetaiotaomicron* can utilize xenosiderophores enterobactin and stealth siderophore salmochelin via the xenosiderophore utilization system (Xus), which has been associated with resilience during colitis in mice (Zhu et al., 2020). *B. thetaiotaomicron* captures enterobactin via the surface-exposed lipoprotein XusB, enhancing its resilience under inflammatory conditions. By binding enterobactin via XusB, it becomes unavailable for sequestration by LCN2. Notably*, S*. Typhimurium can recapture XusB-Ent, thereby exploiting it as an iron reservoir for the pathogen and promoting immune evasion (Spiga et al., 2023). This indicates that iron acquisition and immune evasion via xenosiderophores represent key determinants of gut colonization by both commensal and pathogenic microorganisms. Recent findings indicate that XusB plays an important role in ferrichrome utilization in *B. fragilis*, as XusAB-dependent uptake enables growth on ferrichrome, and that many *Bacteroidota* species can utilize fungal siderophores through related xenosiderophore uptake systems (Silale et al., 2026).

Genes encoding the ferrichrome and coprogen transporters FhuA and FhuE are pseudogenized in serovars that do not colonize the intestinal tract, suggesting that fungal siderophore receptors contribute to intestinal infection by non-typhoidal *Salmonella* (Rollenhagen and Bumann, 2006; Nuccio and Bäumler, 2014). Recently, Santus et al. demonstrated that ferrichrome and coprogen, similar to salmochelin, confer a competitive advantage for *Salmonella* in mice, particularly during colitis. They found that the gut mycobiota composition was affected by the diet and detected fungal siderophores in the mouse chow. Siderophore-producing fungi, as well as diet-derived fungal siderophores, promoted *Salmonella* growth, and provided a competitive advantage over Δ*fhuA*/Δ*fhuE* deletion strains, indicating the utilization of fungal hydroxamate siderophores. These data provide the first evidence that fungal siderophores can be obtained through the diet and utilized by gut bacteria (Santus et al., 2022).

In general, the secretion of secondary metabolites by bacteria and fungi affects interactions in microbial communities. For instance, compared to bacteria, fungi contribute the majority of secondary metabolites in the microbiome of cheese rinds, a finding that is likely to apply also to other environmental conditions (Luu et al., 2023). Specifically, cheese-associated fungi, as well as skin- and soil-derived fungal species, modulate bacterial iron accessibility through the secretion of ferrichrome and coprogen, thereby influencing bacterial fitness (Pierce et al., 2021).

Common respiratory tract colonizers, such as *Streptococcus pneumoniae, S. aureus*, and *Haemophilus influenzae*, produce functional hydroxamate siderophore transporters, and the uptake of ferrichrome by these bacteria has been demonstrated (Sebulsky et al., 2000; Pramanik and Braun, 2006; Morton et al., 2010). The hydroxamate transporter represents the only siderophore uptake pathway identified in *H. influenzae* to date and the species is unable to use other siderophores such as enterobactin, aerobactin, and desferrioxamine. In *P. aeruginosa*, the ferrichrome transporter is involved in biofilm formation, elastase activity, and infectivity (Lee et al., 2016). In murine models, administration of ferrichrome abrogated the LCN2-mediated defense against *E. coli* infection in the lungs, leading to a significant rise in lethality (Wu et al., 2010). These results offer mechanistic evidence that ferrichrome could contribute to bacterial immune evasion within the respiratory tract.

Beyond evasion of LCN2, ferrichrome and other siderophores have been shown to directly modulate host immune responses, further supporting an immune-evasive role (Paauw et al., 2009; Saha et al., 2017). In particular, ferrichrome inhibited neutrophil activity and exerted immunosuppressive effects on T cells in mice, presumably by chelation of iron (Saha et al., 2017).

Ferrichrome may also contribute to co-infections caused by bacteria and pathogenic fungi. In a biofilm co-culture of *P. aeruginosa* and *A. fumigatus*, ferrichrome and related fungal siderophores were identified, suggesting the presence of ferrichrome during co-infection of bacteria and fungi in cystic fibrosis (Bastos et al., 2022).

Together, these reports highlight the significant role of ferrichrome as a xenosiderophore in the context of host-pathogen interactions. Evasion of LCN2, support of bacterial iron uptake, and immune modulation through its iron chelation activity contribute to bacterial virulence and fitness. Uptake and utilization of diet-derived fungal siderophores by gut bacteria suggest that ferrichrome plays an important role for pathogenic and commensal bacteria.

### 2.3 The FhuA ferrichrome receptor as entry point for bacteriophages and toxins

The ferrichrome receptor FhuA has been identified as the receptor used by several bacteriophages for cell entry, most prominently the coliphages T1, T5, and phi80 (Hantke and Braun, 1975; Luckey et al., 1975; Wayne and Neilands, 1975). The attachment of phages T1 and phi80 is FhuA- and TonB-dependent, while T5 infection occurs independently of TonB (Braun et al., 1973). Recent studies have identified novel coliphages (JLBYU37, JLBYU41, JLBYU60, Teewinot, Sp15) that exploit FhuA as an entry point (Purnell et al., 2022; Lewis et al., 2023; Azam et al., 2024). The receptor is also implicated in the development of bacterial resistance to bacteriophages, although understanding of phage resistance mechanisms remains limited (Li et al., 2019; Nang et al., 2024).

The interaction of bacteriophage T5 with the FhuA receptor has been utilized to characterize FhuA functionality, particularly to investigate its role in ferrichrome translocation and to test the susceptibility to siderophore-antibiotic conjugates (SACs) (Brochu et al., 1992; Locher and Rosenbusch, 1997; Killmann et al., 1998). Recently, the interaction of T5 phages with FhuA has been exploited for endolysin-engineering strategies to design novel antibacterial therapeutics (Zampara et al., 2020).

Other natural compounds utilizing ferrichrome receptors as a point of entry include albomycin, microcin J25, colicin M, and certain pyocines (Braun et al., 1973; Hartmann et al., 1979; Salomón and Farías, 1993; Ghequire et al., 2017).

The use of the ferrichrome uptake machinery by bacteriophages and natural antimicrobial peptides as an entry route can be exploited for the development of novel antimicrobial agents.

## 3 Chemical synthesis of ferrichrome

### 3.1 Ferrichrome structure

Ferrichrome is one of the best-characterized hydroxamate siderophores and exhibits a highly distinctive architecture. It is a molecule composed of six amino acids: three glycines and three hydroxyornithines (Miller, 1989). The glycine residues serve as “linker elements,” conferring conformational flexibility, whereas the modified ornithines are responsible for iron chelation; each contributes a hydroxamate group that coordinates Fe(III) in an octahedral geometry (Figure 2) (Miller, 1989; Al Shaer et al., 2020).

**Figure 2:**
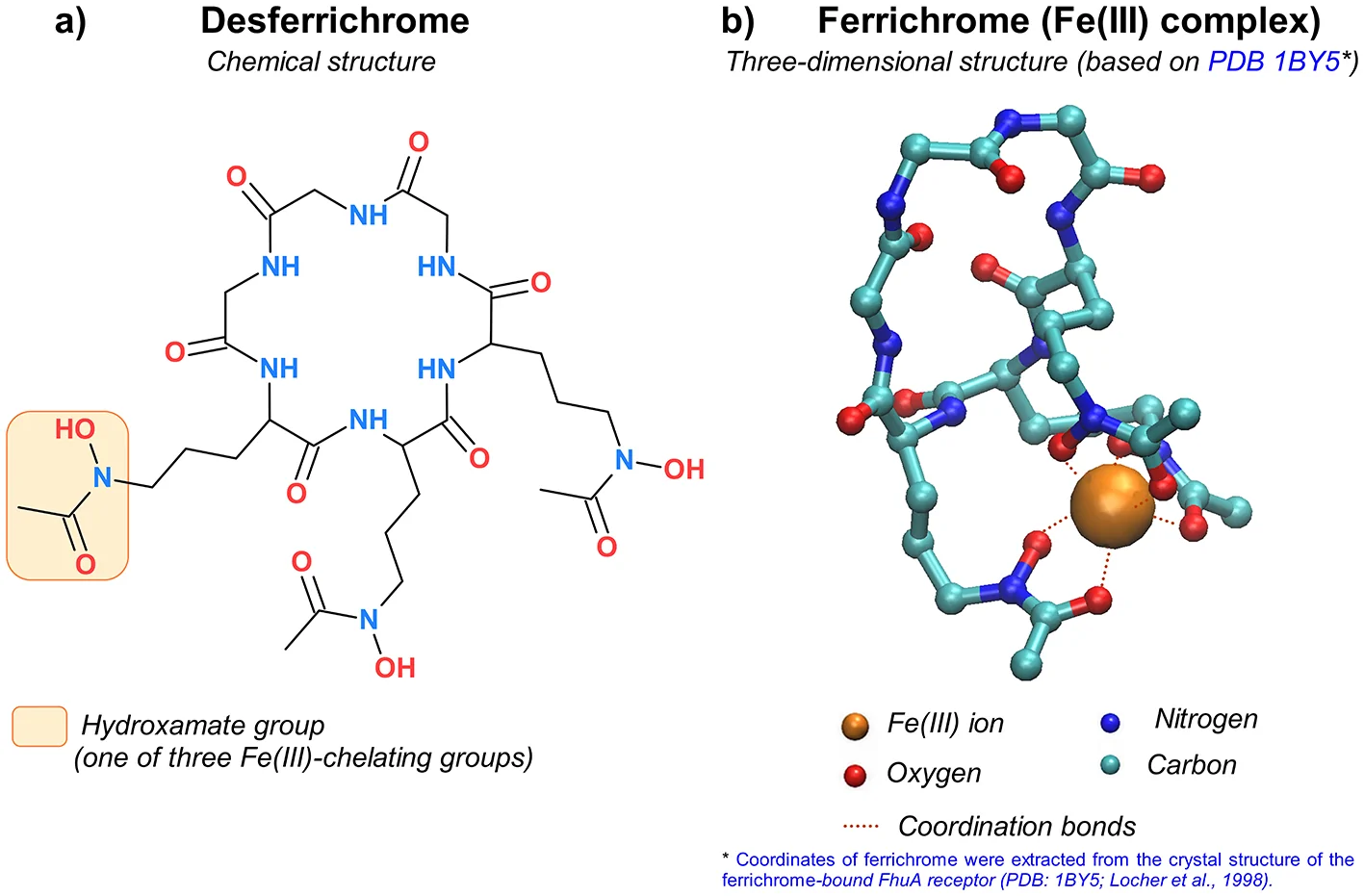
**(A)** Chemical structure of desferrichrome. The gray box highlights one of the N^5^-acetyl-N^5^-hydroxy-L-ornithine residues responsible for iron coordination. **(B)** Three-dimensional structure of Fe(III)-complexed ferrichrome visualized using VMD (Humphrey et al., 1996). Ferrichrome coordinates were extracted from the crystal structure of the ferrichrome-bound FhuA receptor (PDB: 1BY5; Locher et al., 1998). Fe(III) is coordinated by six oxygen donor atoms from three hydroxamate groups; the coordination bonds are indicated by dotted lines. The Fe(III) ion is shown in orange, oxygen atoms in red, nitrogen atoms in blue, and carbon atoms in cyan.

This amino acid composition has important implications for synthetic design. The simplicity of glycine residues makes them straightforward to introduce synthetically and free from stereochemical complications, which renders the linear backbone relatively accessible. By contrast, N-hydroxyornithine represents the main synthetic challenge. Its preparation requires access to suitably protected derivatives (Dolence et al., 1991; Vasanthakumar and Suresh Babu, 2003; Jiang et al., 2006) and a carefully controlled introduction of the hydroxamate functionality, while preserving the integrity of the peptide backbone and orthogonal protecting groups (Miller, 1989; Al Shaer et al., 2020). The cyclic nature of ferrichrome further complicates the synthesis, as the ring-closing reaction must occur selectively without producing polymeric by-products (Fang et al., 2024; He et al., 2024).

In practice, the peptide-based framework of ferrichrome enables the use of diverse cyclization strategies, while modifications to the hydroxyornithine units enable the design of ferrichrome mimics—molecules that retain Fe(III)-chelating ability but feature simplified or more stable scaffolds. It is also important to distinguish between ferrichrome and desferrichrome—the latter represents the iron-free form (Leong and Raymond, 1974). In most synthetic approaches, the iron-free precursor is prepared first and subsequently subjected to Fe(III) complexation to regenerate the native siderophore. This strategy avoids complications related to the potential instability of hydroxamate groups in the presence of transition metals during peptide assembly.

### 3.2 Natural analogs from the ferrichrome-type family

The family of ferrichrome-type siderophores includes a range of structurally related iron chelators built on a cyclic oligopeptide scaffold (Figure 3). In most cases, these are hexapeptides coordinating Fe(III); however, there are exceptions with a different number of residues, such as octapeptides (Koulman et al., 2012) or heptapeptides (Deml et al., 1984). Differences between analogs arise from (i) variations in the amino acid composition of the peptide core (e.g., replacement of Gly by Ser) (Jenner et al., 2023) and (ii) the type and stereochemistry of acylation at the N^5^-hydroxyornithine residues (e.g., anhydromevalonyl derivatives) (Haas, 2014), which modulate Fe(III) affinity, stability, and recognition by transporters (Jenner et al., 2023).

**Figure 3:**
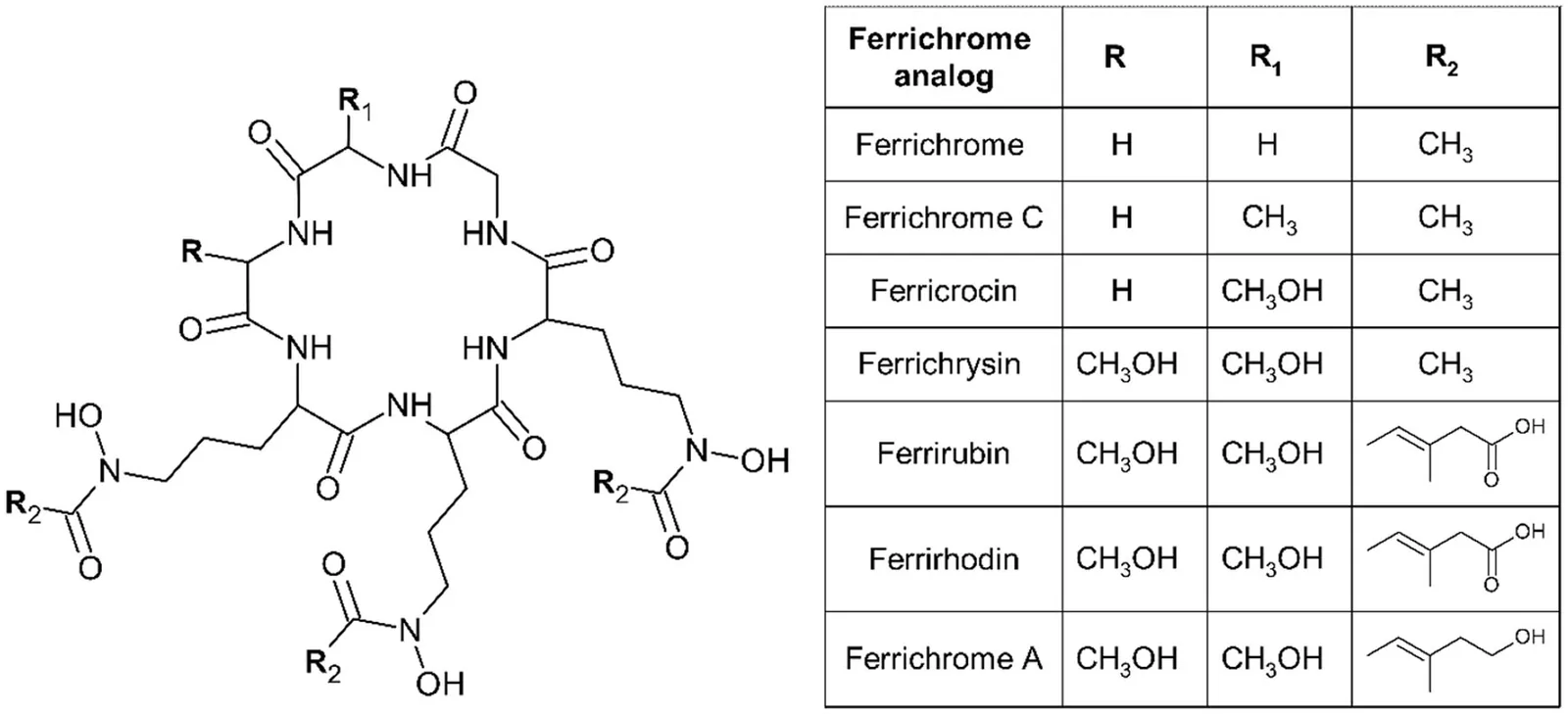
General chemical scaffold of ferrichrome analogs **(left)** and representative natural variants with corresponding substituents **(right)**. Structural differences arise from variations in the peptide core (R and R_1_) and the acylation pattern of N^5^-hydroxyornithine residues (R_2_).

A well-characterized example of a natural ferrichrome analog is ferricrocin, a cyclic hexapeptide with the sequence Gly-Ser-Gly-(N^5^-acetyl-N^5^-hydroxyornithine)_3_. It functions as an Fe(III) storage and chelation system in many fungi and is often used as a model compound to study iron uptake pathways (Schrettl et al., 2004). Related compounds—ferrichrysin, ferrirubin, and ferrirhodin—are also hexapeptides in which the core contains Ser-Ser-Gly instead of Gly-Ser-Gly. The critical differences between them lie in the acylation pattern of hydroxyornithines. Ferrirubin and ferrirhodin are isomers that differ in the stereochemistry of their anhydromevalonyl substituents (*trans* vs. *cis*), which affects conformation and metal-binding properties (Fidelis et al., 1990; Will et al., 2024).

Another variant is ferrichrome A, produced, among others, by *Ustilago maydis*. It differs from ferrichrome in the nature of the acyl substituents attached to the N^5^-hydroxy groups of all three ornithine residues. These acyl chains—primarily *trans*-β-methylglutaconyl groups—are more elaborate than the acetyl groups of classical ferrichrome and introduce additional stereogenic centers (Yuan et al., 2001).

Overall, the “ferrichrome family” represents a set of structurally well-defined yet functionally diverse analogs. Subtle variations—such as Ser↔Gly substitutions in the core, *cis*/*trans* acylation stereochemistry, or changes in ring size—profoundly influence Fe(III) chelation parameters and transmembrane transport properties (Jenner et al., 2023). The cellular roles and functional characteristics of representative natural members of the ferrichrome family are summarized in Table 2. These structural variations not only affect Fe(III) coordination and recognition by bacterial transport systems but also determine the suitability of ferrichrome-type scaffolds for further functionalization and biomedical applications, as discussed in the following sections.

**Table 2:** Representative natural members of the ferrichrome family.

Compound	Producing organism(s)	Peptide core (positions 1–3)	N^5^-Acyl substituent on the three hydroxyornith -ines	Biological role/experimental context	Structural/ functional feature	References
Ferrichrome	*Ustilago sphaerogena;* also *U. maydis, Aspergillus spp*. and *Penicillium spp*.	Gly–Gly–Gly	Acetyl	Fe(III) acquisition; active cellular uptake demonstrated in *U. sphaerogena*. Utilized as a xenosiderophore by bacteria, including *E. coli* K-12 and *S*. Typhimurium *LT-2*.	Prototypical and extensively characterized member; recognized by specific outer-membrane ferrichrome receptors in enterobacteria.	Neilands, 1952; Van Der Helm et al., 1980; Constantine et al., 1990
Ferrichrome A	*Ustilago maydis*	Ser–Ser–Gly	*trans*-β-methylglutaconyl	Fe(III) acquisition; extracellular siderophore produced by *U. maydis* in response to iron starvation.	Ferrichrome-family cyclic hexapeptide; differs from ferrichrome in the neutral amino-acid core and N^5^-acyl substituents.	Yuan et al., 2001
Ferricrocin	*Aspergillus fumigatus*	Gly–Ser–Gly	Acetyl	Intracellular iron storage/homeostasis; ferricrocin supplementation rescues growth and conidiation defects of siderophore-deficient *A. fumigatus*.	Intracellular ferrichrome-type siderophore; production is strongly induced by iron starvation.	Schrettl et al., 2004
Ferrichrysin	*Aspergillus spp*.; *Penicillium spp*.	Ser–Ser–Gly	Acetyl	Fe(III) acquisition; ferric ferrichrysin is utilized as a xenosiderophore by *Candida albicans* via the CaSit1/CaArn1 transporter.	Two Gly → Ser substitutions; same acyl chemistry as ferrichrome	Renshaw et al., 2002; Winkelmann, 2007; Szebesczyk et al., 2016
Ferrirubin	*Aspergillus ochraceus*	Ser–Ser–Gly	*trans*-anhydromevalonyl	Fe(III) acquisition; functions as a xenosiderophore in *P. aeruginosa*, where ferrirubin-mediated iron uptake was demonstrated by growth and ^55^Fe incorporation assays and involves the FpvB transporter.	Ferrichrome-type siderophore; *trans* stereoisomer of ferrirhodin. Uptake is strongly species-dependent and was reported to be poor in *N. crassa*.	Fidelis et al., 1990; Krasulova et al., 2024; Will et al., 2024; Dvorakova Bendova et al., 2025
Ferrirhodin	*Aspergillus versicolor, A. nidulans, A. oryzae, Botrytis cinerea, Fusarium sacchari*	Ser–Ser–Gly	*cis*-anhydromevalonyl	Fe(III) acquisition; efficient iron uptake demonstrated in *N. crassa* and xenosiderophore-mediated iron uptake in *P. aeruginosa* via FpvB.	Ferrichrome-type siderophore; *cis* stereoisomer of ferrirubin. Stereochemistry strongly influences transporter recognition and uptake efficiency.	Fidelis et al., 1990; Krasulova et al., 2024; Will et al., 2024

The structures of the compounds are presented in Figure 3. Ring-size variants that exist outside the canonical hexapeptide scaffold, include octapeptide ferrichrome-type analogs (Koulman et al., 2012) and heptapeptide variants (Deml et al., 1984).

### 3.3 Historical and modern strategies for ferrichrome chemical synthesis

#### 3.3.1 Early solution-phase syntheses

The first attempts to synthesize ferrichrome relied on classical solution-phase peptide methods and marked a significant milestone in understanding both the structure and function of this siderophore (Rogers and Neilands, 1964; Miller, 1989). A pioneering achievement was reported by Keller-Schierlein and Maurer in the late 1960s, in which ferrichrome and its analogs were obtained via stepwise synthesis of a linear hexapeptide composed of glycine and ornithine residues protected with nitro groups (Keller-Schierlein and Maurer, 1969; Maurer and Keller-Schierlein, 1969; Kobayashi et al., 2013). After peptide chain cyclization, the nitro groups were reduced to hydroxylamines and subsequently acylated, thereby reconstructing the hydroxamate moieties critical for iron binding. Although the overall yield was low (approximately 10%), the study represented a landmark proof-of-concept, demonstrating the feasibility of synthetic ferrichrome and providing key insights into functional group protection and deprotection strategies (Miller, 1989).

Another major contribution came from Isowa, Ohmori, Kurita, and colleagues, who reported the total synthesis of ferrichrome in 1974 (Isowa et al., 1974). Their strategy also employed solution-phase peptide synthesis, beginning with assembly of a linear hexapeptide consisting of glycine and ornithine residues. Using carboxyl-activating reagents, they achieved successful macrocyclization, followed by selective deprotection and acylation of hydroxylamine groups. This methodology enabled the construction of structurally correct ferrichrome, whose Fe(III) chelating activity was confirmed (Isowa et al., 1974).

Despite these breakthroughs, early solution-phase syntheses faced substantial challenges. The need for multiple protecting groups, laborious deprotection and functionalization steps, and particularly the problematic macrocyclization often resulted in oligomeric by-products. Moreover, the incorporation of hydroxamate groups was technically demanding, as they are sensitive to degradation and difficult to control chemically. As a result, yields were generally low, and synthetic routes were impractical for large-scale applications (Rogers and Neilands, 1964; Isowa et al., 1974; Miller, 1989). These limitations underscored the necessity for more efficient strategies, ultimately paving the way for solid-phase peptide synthesis (SPPS) and the design of simplified ferrichrome mimics with comparable biological properties.

#### 3.3.2 Application of SPPS

The introduction of SPPS revolutionized ferrichrome synthesis by addressing many limitations of solution-phase methods. In SPPS, all peptide elongation and modification steps occur on a solid support, which simplifies purification, reduces side reactions, and enhances overall efficiency.

Kobayashi and co-workers developed a key building block: Fmoc-Orn(Ac,Ot-Bu)-OH, an Nα-Fmoc-protected N^5^-acetyl-N^5^-hydroxyornithine carrying a *t*-Bu protecting group (Kobayashi et al., 2012). This monomer enabled standard Fmoc-SPPS on 2-chlorotrityl (2-Cl-trityl) resin. The linear precursor was assembled using conventional activation with DIC/HOBt (*N, N*′-diisopropylcarbodiimide/1-Hydroxybenzotriazole). Following resin cleavage, macrocyclization was performed with diphenylphosphoryl azide (DPPA) in the presence of NaHCO_3_, affording the cyclic scaffold. Final t-Bu deprotection revealed the hydroxamate functionalities, yielding desferriferrichrysin (the iron-free cyclic hexapeptide), which could subsequently be complexed with Fe(III). In the same line of work, synthesis of desferriferrichrome was also reported, where serine residues were replaced by glycines. As with desferriferrichrysin, the peptide was synthesized in iron-free form by SPPS and complexed with Fe(III) only at the final stage to afford the biologically active siderophore (Cherkupally et al., 2015; Lin et al., 2018). Notably, these synthesized products were also investigated as pH-responsive indicators, exploiting the Fe(III)-chelating properties of the hydroxamate scaffold to signal changes in protonation state (Kobayashi et al., 2013). These investigations highlighted the potential of ferrichrome and its analogs to function not only as iron-transport agents but also as versatile biochemical and analytical tools.

Recent developments have considerably improved access to functional hydroxyornithine derivatives for SPPS. A particularly useful commercially available building block is Nα-Fmoc-N^5^-acetyl-N^5^-(benzoyloxy)-L-ornithine (Fmoc-Orn(Ac,OBz)-OH) (Cherkupally et al., 2015; Lin et al., 2018). The Obz protecting group is stable under SPPS conditions yet can be selectively removed under mild, orthogonal conditions (1 M LiOH in MeOH/tetrahydrofuran), unveiling the hydroxamate without affecting the peptide backbone. This orthogonality facilitates the precise installation of hydroxamate groups at later synthetic stages. Such strategies have been applied in the synthesis of marine siderophores and extended to studies of ferrichrome analogs, underlining the value of orthogonally protected Orn derivatives in designing siderophore scaffolds and therapeutic mimics (Cherkupally et al., 2015; Tsylents et al., 2026). A comparison of historical and modern ferrichrome synthesis strategies is given in Table 3. The evolution of synthetic strategies for desferrichrome and hydroxamate siderophore scaffolds is presented in Figure 4, outlining the key building blocks and principal synthetic steps.

**Table 3:** Comparison of representative synthetic strategies for ferrichrome-type and hydroxamate siderophores.

Strategy	Key features	Advantages	Limitations	References
Early solution-phase synthesis (1960s)	Nitro-protected hydroxylamine precursor; solution-phase peptide assembly and macrocyclization	Proof of concept: established the feasibility of synthetic ferrichrome	Low yield; multiple protection/deprotection steps; difficult macrocyclization	Rogers and Neilands, 1964; Miller, 1989; Kobayashi et al., 2013
Improved solution-phase synthesis–1974	Preformed protected hydroxylamine followed by macrocyclization, stepwise deprotection, and acetylation	Confirmed the feasibility of synthetic ferrichrome	Laborious; limited scalability	Isowa et al., 1974
SPPS-based synthesis–2012	Fmoc-SPPS with protected Orn building block; solution-phase macrocyclization after resin cleavage	Higher efficiency; simplified purification; modular design	Requires specialized building blocks	Kobayashi et al., 2012
Modern orthogonal SPPS strategies–2015 onward	Orthogonally protected hydroxyornithine building blocks; selective hydroxamate deprotection compatible with peptide synthesis	Precise functionalization; flexibility for analog design	Cost of reagents; synthetic optimization required	Cherkupally et al., 2015; Lin et al., 2018; Tsylents et al., 2024

Key building blocks and principal synthetic steps are illustrated schematically in Figure 4.

**Figure 4:**
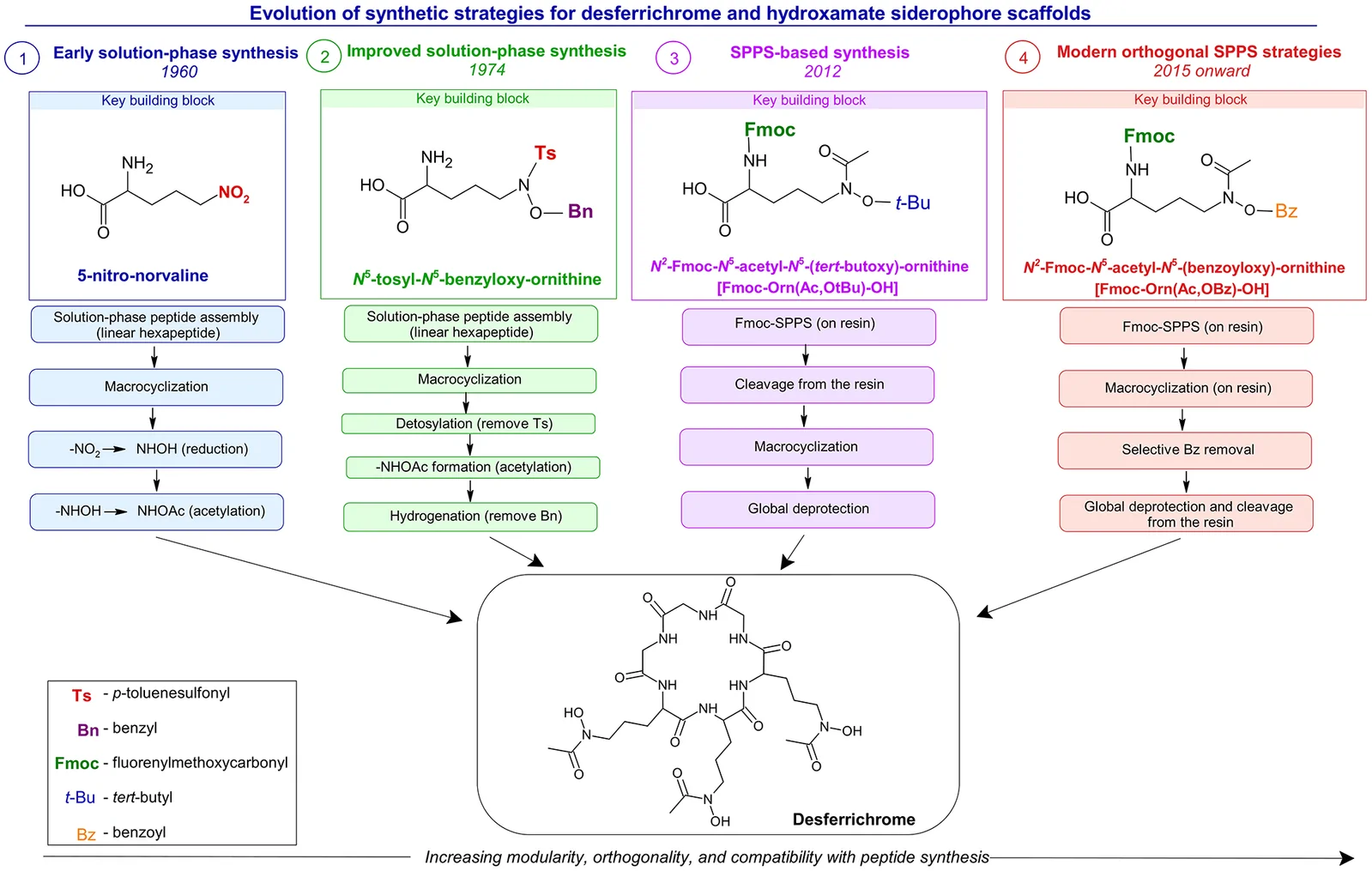
Evolution of synthetic strategies for desferrichrome and hydroxamate siderophore scaffolds. Schematic comparison of representative approaches from early solution-phase synthesis to modern solid-phase strategies. Key building blocks and principal synthetic steps characteristic of each approach are highlighted. The schemes are simplified to emphasize the conceptual development of siderophore synthesis; detailed features, advantages, limitations, and representative references are summarized in Table 3.

Other studies have examined linear ferrichrome derivatives containing hydroxyornithine residues in the context of siderophore biological function, iron chelation and transport (Cezard et al., 2015; Tsylents et al., 2024, 2026). These works emphasized that the availability of orthogonally protected hydroxyornithine building blocks greatly facilitates structural investigations of siderophores and supports the design of therapeutic analogs.

The synthesis of ferrichrome and ferrichrysin via SPPS has significantly simplified the overall process and markedly improved its efficiency compared to classical solution-phase strategies. The application of standard protecting group systems (Fmoc/*t*-Bu, Fmoc/Obz) for hydroxyornithines has broadened interest among research groups in hydroxamate-based siderophores. Current synthetic methodologies not only allow for the faithful reconstruction of native members of the “ferrichrome family” but also enable the introduction of targeted modifications to study their impact on molecular activity.

#### 3.3.3 Specificity of bacterial uptake and related challenges

Ferrichrome and other members of the ferrichrome-type siderophore family are actively transported into bacterial cells via specialized iron uptake systems. In Gram-negative bacteria, the key players are outer membrane proteins belonging to the family of TonB-dependent receptors, which recognize siderophores with high selectivity (Noinaj et al., 2010). A well-known example is FhuA in *E. coli* and related enteric bacteria, which binds ferrichrome and its closely related analogs and, with the help of the TonB–ExbB–ExbD complex, transports them into the periplasm (Braun and Braun, 2002; Ferguson and Deisenhofer, 2002; Braun, 2009; Braun et al., 2023).

The specificity of siderophore–receptor interactions primarily arises from the recognition of the N^5^-acyl-N^5^-hydroxyornithine hydroxamate pattern, which forms a characteristic Fe(III) complex. Substitutions within the peptide backbone (e.g., Gly → Ser) or changes in the stereochemistry of acylation can significantly influence receptor affinity. Studies on ferrirubin and ferrirhodin demonstrated that, despite their similar ability to chelate Fe(III), only some of these analogs are effectively transported by FhuA, highlighting the importance of subtle conformational differences in receptor recognition (Fidelis et al., 1990; Will et al., 2024).

Beyond chemical recognition of the hydroxamate pattern, spatial complementarity between the siderophore and the receptor binding pocket is essential (Sheldon and Heinrichs, 2015). One of the main challenges in designing ferrichrome analogs is that even minor structural modifications can strongly reduce transport efficiency (Tsylents et al., 2024). On the one hand, such modifications are desirable to improve stability or pharmacological properties; on the other hand, they may result in a loss of recognition by bacterial receptors. This tight coupling between siderophore structure and receptor recognition effectively restricts which structural variants can be actively transported, creating a major challenge for the design of therapeutic analogs—for example, siderophores conjugated with drugs (the so-called “Trojan horse” strategy). Preserving the critical recognition features while enabling feasible chemical synthesis and drug conjugation is therefore essential for the success of this approach.

In summary, the specificity of ferrichrome and its analogs in bacterial uptake represents both a great advantage (selective delivery into bacterial cells) and a significant limitation (high sensitivity to structural modifications). Understanding the molecular determinants of siderophore–receptor recognition remains crucial for harnessing this class of compounds in the development of modern antibacterial therapies.

## 4 Ferrichrome as a carrier for antimicrobial compounds

Natural sideromycins, such as albomycin, inspired the development of synthetic SACs, with cefiderocol representing the first clinically approved SAC (McCreary et al., 2021). The current state of research on SACs has been reviewed recently, specifically addressing the limitations and advantages of different siderophores (Miao et al., 2025; Ni and Liu, 2025). Design principles for SACs are discussed in a publication by Luo and Peczuh (2024). Here, we give an overview of SACs involving ferrichrome or ferrichrome analogs and summarize current data about resistance development against these new antibacterials. A summary of the major biomedical applications of ferrichrome is presented in Figure 5.

**Figure 5:**
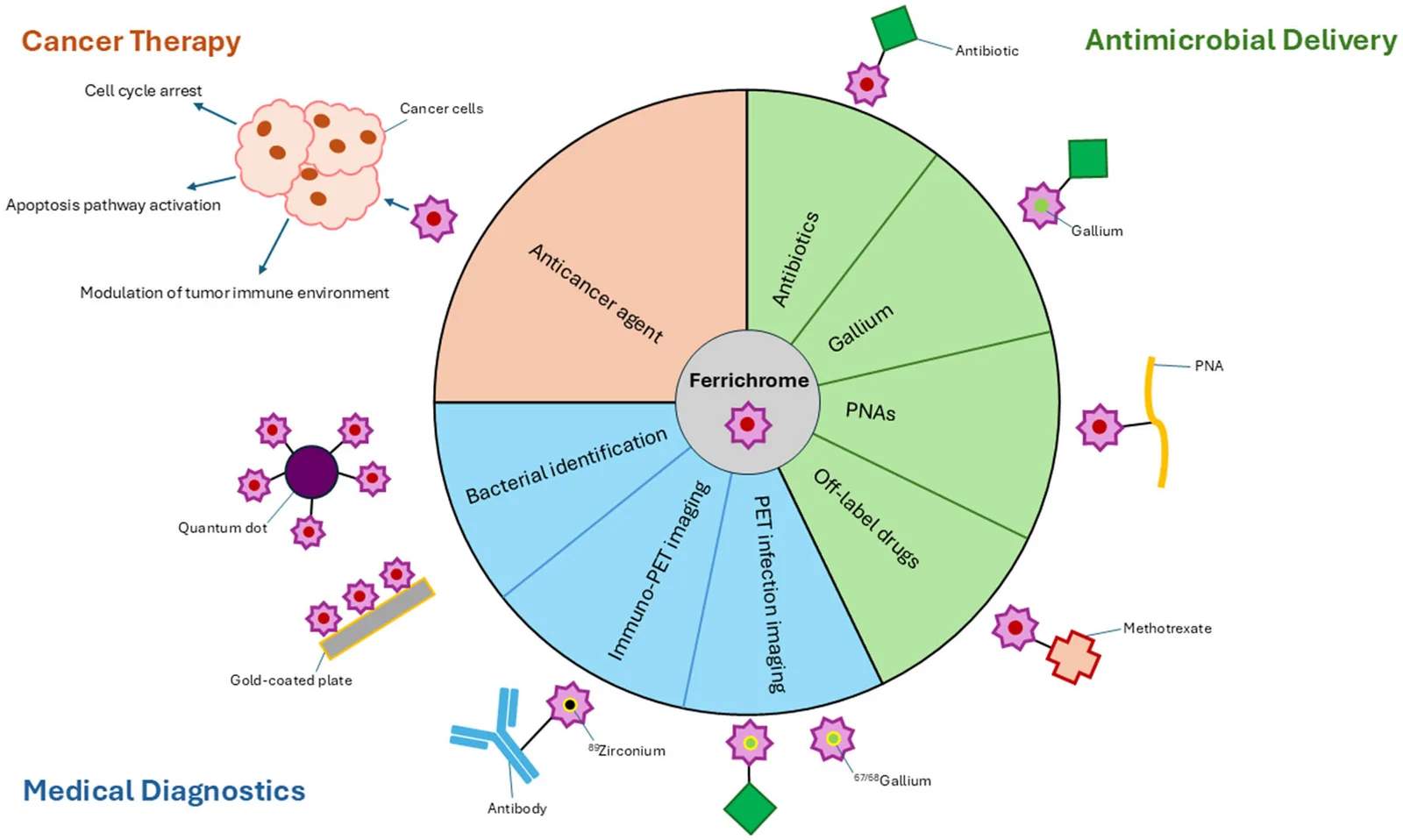
Schematic overview of the major biomedical applications of ferrichrome and representative examples of ferrichrome-associated compounds, conjugates, and biological interactions. Colors indicate the major application categories (green = antimicrobial applications, blue = medical diagnostics, red = cancer-related applications). Chemical structures and symbols are simplified and not shown to scale.

Albomycin, the first natural sideromycin to be identified, comprises a linear trihydroxamate formed by three linked N^5^-acetyl-N^5^-hydroxyornithine residues, covalently attached to an antibacterial thioribosyl pyrimidine moiety. These components are connected via a cleavable serine linker that is hydrolyzed intracellularly by a peptidase. The released antibiotic ‘warhead' subsequently inhibits seryl-tRNA synthetase, leading to cell death (Hartmann et al., 1979; Braun et al., 1983). Albomycin was first isolated from *Streptomyces griseus* in 1947 (Reynolds et al., 1947) and is also produced by *Streptomyces globisporus* and *Actinomyces subtropicus* (Gause, 1955; Zakalyukina et al., 2022). The three albomycin congeners, δ_1_, δ_2_, and ε, differ in the nature of their pyrimidine base and N^4^ substituents: the δ congeners contain a 2-thiocytosine moiety and differ from each other in their N^4^-acyl groups, while the ε congener bears a cytosine base, with δ_2_ being the best studied and most potent antibacterial variant (Benz et al., 1982; Lin et al., 2018). Albomycin has been instrumental in characterizing hydroxamate TonB-dependent transport systems in *E. coli* and *S. pneumoniae* and exhibits effective antibacterial activity against both Gram-negative and Gram-positive bacteria, including *E. coli, S. pneumoniae*, and *S. aureus*. MIC values of albomycin in these bacteria ranged from low ng/mL to low μg/mL (Gause, 1955; Pramanik et al., 2007; Lin et al., 2018). In *S. aureus* and *S. pneumoniae* clinical isolates, albomycin δ_2_ showed superior efficacy compared to ciprofloxacin, vancomycin, and penicillin G (Lin et al., 2018).

Despite promising efficacy and tolerability in animal models, clinical development of albomycin has been impeded primarily by challenges in large-scale production. Bioengineering approaches using *S. griseus* have improved biosynthetic yields but remain suboptimal (Zeng et al., 2012; Kulkarni et al., 2016). Recent advances in understanding the biosynthetic pathway of albomycin may help to optimize this approach (Ushimaru et al., 2020; Li et al., 2023). Although partial synthetic routes were reported earlier (Fiedler et al., 1985; Bredenkamp et al., 1991), a complete and efficient total synthesis of albomycin was only achieved in 2018 (Lin et al., 2018; Gadakh et al., 2020).

In pioneering works by Miller's group, a method to synthesize tri-δ-N-hydroxy-L-ornithine peptide siderophores, designed to mimic ferrichrome, was established and conjugates with antibiotics, such as loracarbef, oxamazin, and virginiamycin, were created (Dolence et al., 1991; Lin et al., 1999). The ferrichrome mimics and their antibiotic conjugates were transported into bacterial cells. However, the conjugates demonstrated only limited antimicrobial activity, with significant effects observed only in β-lactam-hypersensitive *E. coli* X580 (Brochu et al., 1992; Minnick et al., 1992; Lin et al., 1999). During antibacterial testing of the loracarbef conjugate, resistant mutants emerged. Resistance was mainly attributed to a disrupted expression of the FhuA protein (Brochu et al., 1992; Minnick et al., 1992).

Modified aminoacyl tRNA synthetase (aaRS) inhibitors with low antibacterial activity were coupled to the siderophore transport module of albomycin. The compounds showed effective inhibition of aaRS in cell extracts but lacked antibacterial activity. The authors proposed that inefficient bacterial uptake of the conjugates may have accounted for the lack of antibacterial activity (Vondenhoff et al., 2012).

### 4.1 Ga-complexed siderophore-antibiotic conjugates

Gallium has demonstrated antibacterial activity against a broad spectrum of bacterial species by chemically mimicking Fe(III), while remaining redox-inert under physiological conditions, thereby disrupting iron-dependent metabolic pathways (Choi et al., 2024). The Boros laboratory substituted iron with gallium in SACs to enhance antibacterial activity. Inspired by albomycin and salmycin, a series of synthetic linear desferrichrome (LDFC) and desferrioxamine (DFO) analogs were generated and conjugated with ciprofloxacin. Here, DFO served as a commercially available surrogate for danoxamine, the native trihydroxamate siderophore of salmycin. In the initial study, the antimicrobial activity of Ga(III) complexes of the LDFC-ciprofloxacin (D2) conjugate, containing a non-cleavable β-alanine linker, was investigated and compared with iron-bound (Fe) and apo-variants (Pandey et al., 2019). Ga-D2 showed broad-spectrum activity against *E. coli, S. aureus, P. aeruginosa*, and carbapenem-resistant *Klebsiella pneumoniae*. It demonstrated particular efficacy against *S. aureus*, exhibiting a MIC90 of 0.93 μM, outperforming ciprofloxacin and matching the potency of albomycin. Interestingly, Ga(III)-D2 retained activity in *P. aeruginosa*, whereas albomycin and the iron-complexed variant Fe-D2 were ineffective (Pandey et al., 2019).

The Ga-complexed LDFC-ciprofloxacin conjugate featuring a cleavable serine linker (D5) was termed Galbofloxacin. It exhibited a tenfold increase in potency compared to ciprofloxacin with a MIC98 of 0.93 μM, which positions it among the most active synthetic Trojan Horse antibiotics (THAs) against *S. aureus* to date. In a mouse *S. aureus* infection model, Ga(III)-D5 demonstrated superior efficacy and survival benefit over ciprofloxacin. *In vitro* cytotoxicity assessment in HEK-293 cells indicated no toxicity up to 80 μM and approximately 85% viability at 100 μM, showing a cytotoxicity profile comparable to that of ciprofloxacin (Pandey et al., 2021). In *P. aeruginosa*, the antimicrobial efficacy of Ga(III)-D5 decreased 5-fold compared to Ga(III)-D2, while Fe(III)-D5 unexpectedly led to growth promotion (Pandey et al., 2021).

In contrast to previous studies (Banin et al., 2008; Frangipani et al., 2014), gallium-complexed siderophores alone showed no inhibitory effect (Pandey et al., 2019). Overall, in almost all bacterial strains tested, the iron-complexed ferrichrome–ciprofloxacin conjugates showed the lowest antimicrobial efficacy, while apo-conjugates retained partial activity, and gallium-complexed conjugates were the most effective.

Expression profiles of *S. aureus* treated with Ga(III)-complexed siderophore-ciprofloxacin conjugates revealed a disruption of iron and heme homeostasis. Heme import, staphyloferrin synthesis, and protein synthesis were reduced. In the presence of Fe-complexed conjugates, genes involved in siderophore import and iron storage were downregulated, whereas their expression was maintained upon exposure to Ga(III)-complexed conjugates - likely contributing to the enhanced antimicrobial activity of Ga(III)-complexed variants (Marlin et al., 2025).

Galbofloxacin-resistant *S. aureus* mutants were generated by serial passaging in the presence of sub-MIC concentrations of Galbofloxacin (Marlin et al., 2025). Acquisition of resistance was primarily associated with mutations in siderophore transporter genes and in genes likely related to the mode of action of ciprofloxacin. Mutations in two previously uncharacterized ABC transporters, tentatively implicated in siderophore transport, were associated with Galbofloxacin resistance. Transposon-mediated inactivation of *fhuB* and the ferric siderophore transporter caused diminished potency of Galbofloxacin (Marlin et al., 2025). Together, these findings underscore the critical role of siderophore uptake in mediating antibacterial activity.

The improved antibacterial activity of Ga(III)-complexes over Fe(III)-complexes, attributable to the unperturbed expression of siderophore-transport genes, suggests that gallium represents a broadly applicable strategy for SACs. The gallium-complexed constructs created by the Boros lab represent one of the most well studied and promising new SACs that incorporate ferrichrome. Other metals have recently been explored in this context, such as bismuth incorporated in cefiderocol and ruthenium–siderophore complexes designed for intracellular prodrug activation (Southwell et al., 2023; Wang et al., 2023).

### 4.2 Ferrichrome conjugation for drug repurposing

In a recent study, ferrichrome was employed to repurpose the anticancer drug methotrexate (MTX) as an antibacterial agent (Zhao et al., 2022). MTX inhibits the dihydrofolate reductase (DHFR), thereby disrupting folate metabolism in both mammalian and bacterial cells. To overcome the poor cellular permeability of MTX in bacteria and minimize its toxicity in human cells, the drug was conjugated to a ferrichrome analog via a non-cleavable linker. Several conjugates with linkers differing in length and glutamic acid content were created and tested for antibacterial activity. The most potent conjugate, compound 8, improved the antibacterial activity against *S. pneumoniae* and *Y. enterocolitica* compared to MTX, with MIC values ranging from 1.72 nM to 6.84 nM across *S. pneumoniae* strains, and 6.84 nM in *Y. enterocolitica*. Notably, it was substantially more potent than the conventional antibiotics ampicillin and ciprofloxacin and could be considered among the most potent antibacterial THAs to date. The authors further observed that length, rigidity, hydrophobicity and branching of linkers did not have a noticeable effect on activity, while the addition of three glutamic acid residues drastically decreased activity in *S. pneumoniae*. The conjugation of MTX to ferrichrome dramatically reduced the cytotoxicity of MTX in human liver cells, indicating an improved specificity of MTX toward bacteria. Moreover, the siderophore conjugation enhanced the inhibitory activity of MTX against isolated DHFR from *S. pneumoniae* by 8-fold. The authors hypothesized that the siderophore and linker moieties created additional binding interactions with DHFR (Zhao et al., 2022). A subsequent study with a DFO-MTX conjugate further confirmed the high potency of this strategy, especially against *S. pneumoniae* (Zhou et al., 2025). Together, these results demonstrate that conjugation with ferrichrome can improve the uptake of compounds with poor transport into bacterial cells, thereby facilitating the repurposing of off-label agents into effective antibacterials. However, further *in vivo* studies are required to determine whether ferrichrome conjugation can successfully translate the antibacterial activity of MTX into therapeutic efficacy.

### 4.3 Ferrichrome as a carrier for antibacterial peptide nucleic acids (PNAs)

A linear ferrichrome mimic has been used as a carrier molecule for antibacterial peptide nucleic acids (PNAs) (Tsylents et al., 2024, 2026). PNAs are synthetic DNA analogs bearing a polyamide backbone that can be used as specific antisense oligonucleotides (ASOs) binding the mRNA of essential bacterial genes, thereby causing cell death. However, their efficacy and application are currently limited by their poor internalization into bacteria (El-Fateh et al., 2024). A linear ferrichrome analog was conjugated to PNAs with a non-cleavable linker to improve uptake into *E. coli* (Tsylents et al., 2024, 2026). To ensure constitutive expression of ferrichrome uptake genes independently of iron availability and to eliminate effects of enterobactin-mediated iron acquisition, an *E. coli* Δ*fur* mutant was used. The uptake and activity of the PNA conjugate were evaluated by targeting a fluorescence reporter gene. The siderophore-PNA conjugate achieved significant fluorescence silencing, demonstrating that the ferrichrome analog could effectively mediate translocation of the PNA into the cytoplasm without obstructing the PNA-mRNA binding. Notably, the ferrichrome mimic was recognized by the coprogen-receptor FhuE of *E. coli*. Molecular docking analyses revealed that the FhuE residues contacting the linear ferrichrome mimic largely overlapped with those involved in coprogen binding, indicating that the linear ferrichrome mimic showed structural resemblance to coprogen. A cyclic ferrichrome variant showed poor iron binding capabilities, while the linear molecule adopted a structure resembling natural cyclic ferrichrome upon binding of iron. This study provides the first proof-of-concept for the use of hydroxamate-type siderophores as carrier molecules for PNAs.

In summary, ferrichrome and ferrichrome mimics show potential as carriers for targeted delivery of antimicrobial compounds by hijacking iron acquisition systems. Metal substitution and linker design can affect the antibacterial efficacy of the conjugates. However, resistance can arise through alterations in the siderophore transport pathways, including loss or downregulation of TonB-dependent receptors.

## 5 Development of resistance to ferrichrome uptake

The development of resistance to SACs, frequently mediated by the disruption of siderophore uptake pathways, remains a major concern and has gained further attention following the clinical application of cefiderocol (Lin et al., 2019). The siderophore carrier approach is thought to confer an advantage with respect to resistance development, since mutations affecting siderophore transporters are likely to be accompanied by a loss of bacterial fitness. However, conflicting observations have been reported across studies investigating resistance development.

Under laboratory conditions, spontaneous albomycin-resistant *Y. enterocolitica* mutants occurred at a frequency of 10^−4^ to 10^−5^ (Pramanik et al., 2007). These mutants, as well as the constructed albomycin-resistant *S. pneumoniae* D39T1 mutant, were less competitive in mice compared with the wild-type strain and showed reduced proliferation. Albomycin was effective at clearing infections with wild-type *S. pneumoniae* and *Y. enterocolitica* in mice. While it reduced the bacterial load of albomycin-resistant strains, it was unable to fully clear infections caused by resistant mutants.

Miller and co-workers similarly observed the selection of resistant mutants after treatment of hypersensitive *E. coli* with synthetic ferrichrome-derived β-lactam conjugates. A ferrichrome-related ornithine tripeptide siderophore was conjugated with ciprofloxacin to yield the conjugate EKD-3-88, which was assessed in *E. coli* using agar diffusion tests (Brochu et al., 1992). Resistant mutants arose at an estimated frequency of 3.1 × 10^−5^ and exhibited a loss of FhuA expression. In diffusion chambers implanted in rats, resistant mutants initially exhibited impaired growth but recovered growth and susceptibility to EKD-3-88 after 3 h, indicating that the Fhu system is important for growth and that persistence of FhuA-deficient mutants is unlikely.

A similar conjugate, consisting of an ornithine tripeptide siderophore linked to a carbacephalosporin, was tested in the same *E. coli* strain; only one of twelve resistant mutants exhibited impaired growth under iron-limited conditions *in vitro* (Minnick et al., 1992). The absence of ferrichrome uptake in selected resistant mutants indicated that resistance likely arose from impairment of the uptake pathway. This suggests that the previously observed fitness cost associated with impaired ferrichrome uptake might not be as common, although more rigorous quantitative studies are necessary for a robust evaluation.

Current findings on resistance to the clinically approved catechol sideromycin cefiderocol also provide inconclusive evidence for a relationship of resistance and bacterial fitness loss. Mutations in siderophore transporters have been frequently observed both in serial passaging experiments (Nurjadi et al., 2022; Werth et al., 2022; Kriz et al., 2024) and among cefiderocol-resistant clinical isolates (Malik et al., 2020; Klein et al., 2021; Kocer et al., 2024; Santerre Henriksen et al., 2024; Sastre-Femenia et al., 2025). Additional mechanisms implicated in cefiderocol resistance include β-lactamase production, alterations in penicillin-binding proteins, porin loss and, in some species, efflux-pump overexpression (Salleh, 2025).

Overall, currently reported data suggest a low prevalence of cefiderocol non-susceptibility (Karakonstantis et al., 2024; DeJonge et al., 2026). Evidence from *in vitro* fitness experiments suggests that transporter mutations compromise bacterial fitness in *Stenotrophomonas maltophilia, A. baumannii, K. pneumoniae*, and *P. aeruginosa* (Gill et al., 2021; McElheny et al., 2021; Gill et al., 2022; Galdino et al., 2024). Opposing results have been observed in *K. pneumoniae* and *Enterobacter cloacae* (Nurjadi et al., 2022; Tascini et al., 2024).

It is important to note that experiments investigating resistance development *in vitro* are commonly performed without iron limitation (Gill et al., 2021; Nurjadi et al., 2022; Kriz et al., 2024). Mutants isolated under iron-rich conditions might not accurately represent *in vivo* resistance development and could also influence conclusions regarding bacterial fitness.

Surprisingly, some studies have reported an association between mutations in ferrichrome receptors and reduced cefiderocol susceptibility, implying a previously unrecognized overlap between ferrichrome and cefiderocol uptake pathways that warrants further investigation (Ito et al., 2018; Zhang et al., 2022).

Resistance to ferrichrome-based SACs has been primarily associated with mutations in siderophore transport, often accompanied by impaired growth under iron-limited conditions. However, the associated fitness costs appear to depend substantially on the respective bacterial species, potentially reflecting differences and redundancies in their iron-acquisition pathways, and seem to vary between experimental and clinical contexts.

Uptake via multiple receptors has been suggested as a strategy to circumvent resistance associated with mutations in siderophore receptors. Artificial enterobactin mimics TRENCAM and MECAM have demonstrated uptake via two or three different receptors, respectively, retaining activity even when individual transporters were mutated (Pinkert et al., 2021; Motz et al., 2024). Notably, serial passaging of a MECAM-based SAC selected for sustained resistance through mutation of ExbB, a shared component of the Ton-dependent transport system, rather than an individual siderophore receptor (Pinkert et al., 2021). This suggests that multi-receptor uptake may increase the genetic barrier to resistance.

Further, mixed-ligand SACs have been investigated, with Ghosh et al. creating a mixed biscatecholate–monohydroxamate ligand capable of effectively utilizing FhuA and FiuA as uptake receptors in *E. coli* (Ghosh et al., 1996). While this mixed siderophore has been investigated in follow-up studies and has shown antibacterial efficacy with different antibiotics, its effect on resistance development has not been directly assessed (Wencewicz and Miller, 2013; Ghosh et al., 2020). For ferrichrome-based SACs, these strategies have not yet been investigated. However, a better understanding of ferrichrome and ferrichrome-type siderophore recognition could provide promising opportunities for the development of such constructs. For example, the recent discovery that *P. aeruginosa* effectively takes up ferrichrome via both FpvB and FhuA could provide an opportunity to investigate ferrichrome-based SACs designed for multi-receptor uptake (Chan and Burrows, 2023).

The ferrichrome core structure has shown considerable promise as a carrier for poorly transported antibacterial compounds and is featured in several of the most potent THAs under investigation. The first identified natural SAC, albomycin, which incorporates a ferrichrome-like siderophore, may still hold untapped potential as a modern antibacterial agent.

## 6 Ferrichrome in medical diagnostics

The high selectivity of bacterial uptake systems for specific siderophores, combined with their strong metal-chelating capacity, makes siderophores suitable for use in infection imaging, pathogen detection, and bacterial capture (Fan and Fang, 2021). Radio- and fluorescence labeling served as well-established tools for characterizing and visualizing bacterial siderophore uptake and also constitute the primary approach for applying siderophores in medical imaging (Emery and Hoffer, 1980; Weizman et al., 1996; Ardon et al., 1997, 1998).

Petrik et al. were the first to demonstrate the use of siderophores in PET-based infection imaging, employing ^68^Ga(III)-labeled triacetylfusarinine C (TAFC) and ferricrocin (FC), a member of the ferrichrome siderophore family, to visualize pulmonary *Aspergillus* infections in mice (Petrik et al., 2010). Early-phase clinical trials of ^68^Ga-TAFC and ^68^Ga(III)-DFO for imaging of fungal and bacterial infection have been performed recently or are currently ongoing, although these constructs are not part of the ferrichrome family and are therefore not strictly within the scope of this review (Barrington et al., 2026) (EudraCT number: 2020-002868-31, NCT06105411). ^68^Ga(III)-ferrirubin, a ferrichrome-type siderophore, exhibited promising properties for imaging bacterial infection caused by *S. aureus* and *A. baumannii in vitro* and *in vivo* (Krasulova et al., 2024; Dvorakova Bendova et al., 2025). Across both studies, ferrirubin demonstrated favorable pharmacokinetics and consistent ^68^Ga(III)-mediated accumulation in sites of infection with minimal off-target uptake, establishing its suitability as a targeted PET imaging probe for bacterial infection. In contrast, its stereoisomer ferrirhodin showed an inferior pharmacokinetic profile, demonstrating that small structural variations can affect both the pharmacokinetics and the imaging suitability of siderophore-based PET probes (Krasulova et al., 2024).

The Boros group further investigated ^67^Ga(III)-complexed variants of their ferrichrome-ciprofloxacin constructs. Their findings underscore the potential of Ga(III)–ferrichrome systems as PET imaging and theragnostic agents. ^67^Ga(III)-complexed D2 showed a comparable biodistribution in mice to previously studied radiolabeled siderophore complexes. However, it exhibited rapid *in vivo* metabolism comparable to ^18^F-based infection tracers, indicating the need for further optimization of metabolic stability (Pandey et al., 2019). The second-generation variant, ^67^Galbofloxacin, also exhibited promising pharmacokinetic and radiochemical properties in a *S. aureus* infection mouse model. The tracer demonstrated markedly increased uptake in infected muscle relative to ^67^Ga(III)-citrate, showed rapid renal clearance, and displayed negligible off-target organ accumulation. Together with its previously demonstrated therapeutic efficacy in mice, the compound represents a potent theragnostic agent for bacterial infections (Pandey et al., 2021).

The bacterial siderophore DFO has been firmly established as a radiometal chelator for ^89^Zr in clinical application and is considered the gold-standard for immuno-PET imaging (Heskamp et al., 2017). However, undesirable uptake in bones has been observed in mouse studies, motivating the search for new alternatives (Holland et al., 2010; Abou et al., 2011). Hexa- and octadentate ferrichrome analogs were conjugated with the HER2-targeting antibody trastuzumab and proved suitable for ^89^Zr radiolabeling without affecting the inertness of the complexes (Adams et al., 2017). *In vivo*, the ferrichrome-derived immunoPET probes performed comparably to the standard DFO probe in a mouse model and exhibited improved blood-clearance. The octadentate variant exhibited enhanced stability and kinetic inertness. Conjugation with near-infrared fluorophore silicon rhodamine (SiR) enabled a clear separation of a subcutaneous tumor from surrounding tissue in fluorescence imaging *in vivo* and *ex vivo*, allowing for *in vivo* fluorescence-guided surgical resection of the tumor mass. The probes exhibited biodistribution and PET imaging profiles comparable to those of an analogous DFO conjugate, with no impact on imaging performance attributable to fluorophore conjugation. The successful conjugation of ferrichrome derivatives with antibodies and fluorophores, without compromising complex stability, underscores their potential as versatile dual-modality imaging agents and viable alternatives to the gold-standard ^89^Zr-DFO.

Fluorescence labeling of ferrichrome has been commonly utilized for the visualization of siderophore uptake, but rarely in medical imaging applications. It has been important for the identification of a siderophore recycling mechanism in *P. aeruginosa* (Hannauer et al., 2010). Ferrichrome receptors, along with other siderophore-binding proteins from various bacteria, have been fluorescently labeled and incorporated into a sensor system to identify, discriminate, and quantify apo- and ferric siderophores as well as iron. This could represent a novel approach to assess the prevalence and functional roles of siderophores in both environmental and human contexts, although further validation in complex samples and *in vivo* models is warranted (Kumar et al., 2022).

Siderophores deployed for bacterial identification are generally immobilized on metal-coated surfaces to facilitate binding to corresponding bacterial receptors (Passari et al., 2023). Gold surfaces incorporating iron-complexed artificial trihydroxamate or catechol–hydroxamate hybrid siderophores can capture a broad spectrum of microorganisms, whereby the binding efficiency is strongly influenced by the degree of structural resemblance to bacterial siderophores (Inomata et al., 2024). Subsequent detection is achieved via electrochemical impedance. Due to their broad yet selective recognition by microbial receptors, ferrichrome-type siderophores might be promising candidates for incorporation into these bacterial identification systems.

Ferrichrome has been conjugated to quantum dots (QDs) and used as a new type of bioprobe. Quantum dots are nanoscale semiconductor particles with size-dependent fluorescence properties and are exploited for bacterial detection due to their bright, stable, and tunable optical signals, enabling sensitive and specific quantification of bacteria. The ferrichrome-conjugated QDs could bind *P. putida* and *P. fluorescens* with high sensitivity and exhibited cluster formation, while retaining their fluorescent properties. The formation of clusters could be visualized with UV-vis and fluorescence spectroscopy (Wu et al., 2009). QD-ferrichrome conjugates were utilized for an adhesive and emissive platform with the potential to tether microorganisms possessing a ferrichrome receptor, thereby allowing the separation and detection of bacteria based on siderophore content (Besserglick et al., 2017). Advances in designing ferrichrome analogs with varying degrees of bacterial specificity, from broad-spectrum to species-specific recognition, could further improve their utility in microbial detection (Olshvang et al., 2015). It is important to note that this application is in its early stages. Current investigation are limited in scale and depth, warranting further comprehensive validation.

## 7 Ferrichrome as a potential anticancer agent

Iron plays an important role in carcinogenesis, as iron accumulation is frequently observed in tumors and excess iron levels have been associated with an increased cancer risk and poorer prognosis (Chang et al., 2019; Zhou et al., 2024; De Troy et al., 2025). It has been hypothesized that the high iron levels observed in tumors reflect the elevated iron demand of cancer cells to sustain their accelerated growth and proliferation relative to normal cells (Torti and Torti, 2020). Since tumor cells are strongly dependent on iron for their proliferation, they are believed to be more sensitive to iron depletion than normal cells, which has led to increased research into iron chelators as potential anticancer agents. Among the most extensively studied iron chelators are the natural bacterial compound DFO and the synthetic chelator deferasirox (DFX), both of which have undergone or are currently undergoing evaluation in small-scale clinical trials for anticancer applications (Min, 2019; Wilcox et al., 2022; University Hospital, 2024; Memorial Sloan Kettering Cancer Center., 2025). Comprehensive reviews on iron chelators and siderophores in cancer therapy have been published recently (Ibrahim and O'Sullivan, 2020; Pita-Grisanti et al., 2022).

Ferrichrome has shown potent antitumor activity in a series of preclinical studies, which are discussed below. In the initial study, anticancer activity was observed in the culture supernatant of *Lactobacillus casei*, and a subsequent analysis of its composition identified ferrichrome as the main component responsible for this effect (Konishi et al., 2016). Notably, the authors reported a diminished tumor-suppressive effect of the *L. casei* supernatant upon addition of LCN2. Based on current insights into the recognition mechanism and ligand specificity of LCN2, its interaction with ferrichrome is considered highly improbable (Clifton et al., 2019). Identification and characterization of the specific ferrichrome compound produced by *L. casei* may provide further insight into this observation.

In subsequent studies, ferrichrome showed tumor growth suppressive effects in a variety of cancer cells *in vitro* and in murine xenograft models. When tested on tumor cells of the pancreas, colon, and esophagus, its antiproliferative effect was greater than that of 5-FU or cisplatin, and a synergistic effect with 5-FU was observed (Kita et al., 2020; Iwama et al., 2021; Kunogi et al., 2024). Notably, ferrichrome showed antitumor activity in pancreatic cancer cells resistant to 5-FU (Kita et al., 2020). Ferrichrome demonstrated significant cytotoxic activity against gastrointestinal adenocarcinoma, but its effects were comparatively limited in squamous cell carcinoma (Kunogi et al., 2024). Since only one cell line of squamous cell carcinoma (KYSE70) was tested, more extensive studies are needed to verify this observation. Across multiple *in vivo* mouse studies, ferrichrome demonstrated a more favorable safety profile than 5-FU, showing no detectable organ toxicity or adverse effects even at doses far exceeding therapeutic levels and in aged animal models (Kita et al., 2020; Iwama et al., 2021; Kunogi et al., 2024). Notably, repeated or high-dose administration did not reduce systemic iron levels. In general, the mode of action varied between cell types and cell lines, but the ER stress-responsive-JNK/DDIT3-mediated apoptosis pathway was identified as a common mechanism in the antitumor activity of ferrichrome. Notably, this apoptosis pathway has also been implicated in the activity of DFO and DFX in gastric cancer and osteosarcoma cells (Kim et al., 2016; Xue et al., 2021). However, apoptosis induced by DFO and DFX appears to be primarily triggered by reactive oxygen species (ROS), a mechanism not yet reported in ferrichrome-treated cells. Ferrichrome induced cell-cycle arrest in gastric, pancreatic, and esophageal cancer models, acting through p53-dependent mechanisms in pancreatic cancer cells, but through p53-independent, likely DNA-damage–related pathways in esophageal cancer cells (Ijiri et al., 2017; Kita et al., 2020; Iwama et al., 2021; Kunogi et al., 2024).

Ferrichrome has further exerted strong antitumor effects in pancreatic adenocarcinoma by modulating the tumor immune microenvironment. Through TLR4-dependent signaling, it promoted polarization of pro-inflammatory tumor–associated macrophages (TAM), suppressed immunosuppressive TAMs, increased CD8^+^ T-cell infiltration, and reduced myeloid suppressor cells, collectively resulting in potent tumor growth inhibition (Chaib et al., 2022).

Notably, ferrichrome treatment significantly improved the efficacy of anti-PD-L1 immunotherapy, suggesting a potential utility in immune checkpoint blockade therapy. Surprisingly, ferrichrome alone did not exert an antitumor effect in murine pancreatic cancer cells *in vitro*, contrary to previously discussed findings. However, its growth-inhibitory effect in human colon cancer cell lines was consistent with prior observations (Kita et al., 2020). The authors concluded that the antitumor effect of ferrichrome is primarily caused by the antitumor immune response due to modulation of the immune milieu rather than by direct cytotoxic activity, although partial contribution of direct cytotoxicity could not be excluded.

Interpretation of the ferrichrome antitumor effects is hampered by methodological limitation. Most studies employed xenograft models in nude mice, which do not allow the observation of immune-related effects (Konishi et al., 2016; Ijiri et al., 2017; Kita et al., 2020; Iwama et al., 2021; Kunogi et al., 2024). Together, current data demonstrate antitumor effects of ferrichrome *in vitro* and in murine models, supporting its potential for further investigation in anticancer therapy. Preclinical studies are currently underway to further investigate the potential of ferrichrome as a novel chemotherapeutic agent, particularly in combination with existing treatments (Kunogi et al., 2024).

## 8 Conclusions

Ferrichrome occupies a central position among hydroxamate siderophores. Although fungal in origin, ferrichrome-type siderophores serve as classical xenosiderophores for many bacteria, including pathogens and gut commensals, through the conserved Fhu uptake system. At the same time, the ferrichrome uptake system is an entry route for bacteriophages and natural antimicrobial compounds. This feature is exploited for the translocation of novel antimicrobial therapeutics. Ferrichrome-based Trojan-horse antibiotics span natural sideromycins, β-lactam and quinolone conjugates, and repurposed agents such as methotrexate. While the MICs of sideromycins are comparably low, the efficacy of synthetic conjugates remains limited. Important strategies for the optimization of THAs include the variation of linkers, the rational design of structurally modified ferrichrome analogs, and the selection of suitable antimicrobial moieties, including antisense oligonucleotides such as PNAs.

The substitution of Fe(III) with Ga(III) is a key approach, which decouples delivery from iron-uptake feedback. This strategy enabled the development of Galbofloxacin, one of the most potent synthetic THAs reported against *S. aureus*. ^68^Ga(III)-labeled ferrichrome analogs, particularly ferrirubin, are advancing as PET tracers for imaging of fungal and bacterial infection. ^89^Zr–ferrichrome–antibody constructs offer a credible alternative to the widely established DFO for immuno-PET.

Ferrichrome suppressed tumor growth in a variety of tumor cells. Antitumor activity was detected across pancreatic, gastric, colonic and esophageal cancer cells. Ferrichrome treatment showed synergistic effects with 5-FU and with anti-PD-L1 checkpoint blockade therapy. However, the interpretation of antitumor and immune modulating effects is limited by the predominant use of xenograft models in nude mice. Whether the antitumor activity of ferrichrome involves immune modulation, direct cytotoxicity or distinct cellular pathways remains to be elucidated to determine its potential as an anticancer agent.

Due to their high iron affinity and stability, ferrichrome-type siderophores are interesting for a wide range of scientific and biomedical applications. However, the purification of ferrichromes from biological sources is labor-intensive, expensive, and does not allow the design of ferrichrome variants with altered properties. The development of efficient synthetic methods, particularly SPPS and orthogonally protected hydroxyornithine building blocks, has greatly improved access to natural ferrichromes and structurally modified analogs. This progress now makes systematic studies of the relationship between ferrichrome structure, metal coordination, transporter recognition, and biological activity more feasible. Ferrichrome and its analogs therefore remain attractive scaffolds for antimicrobial delivery, diagnostic imaging, and further exploration in cancer therapy.

## 9 Future perspectives

Several questions remain open before ferrichrome-based systems can be translated more broadly into biomedical applications. One of the most important concerns is the molecular basis of recognition by bacterial siderophore transporters. Even relatively small changes in the siderophore scaffold can alter receptor binding and transport efficiency, while some linear ferrichrome mimics are recognized by transporters different from those used by native ferrichrome. The predicted binding site of a linear ferrichrome mimic in FhuE, for example, showed considerable overlap with the residues involved in coprogen binding. A more detailed understanding of these interactions should help to identify structural modifications that are tolerated without compromising uptake.

Structure-based modeling may become increasingly useful in this context. High-resolution transporter structures, molecular docking, and molecular dynamics simulations can support the design of ferrichrome analogs with improved receptor recognition or altered transporter specificity. AI-assisted approaches may further expand this process by helping to screen larger sets of candidate ligands and prioritize variants for synthesis and biological testing. At present, however, such approaches should be viewed as complementary to experimental studies rather than as a replacement for them.

Another important direction is the development of multifunctional siderophore conjugates. The studies summarized in this review show that ferrichrome-derived carriers can be combined with chemically diverse cargos, including conventional antibiotics, repurposed drugs, metal ions, and antisense PNAs. Future conjugates may benefit from more systematic optimization of the siderophore, linker, metal, and cargo as separate components of the same molecule. Cleavable linkers, combinations of different mechanisms of action, or conjugates recognized by more than one uptake pathway may improve activity while reducing dependence on a single transporter.

Transporter-mediated resistance will remain a major challenge. Loss or reduced expression of siderophore receptors can decrease the activity of ferrichrome-based conjugates, although the fitness cost of such mutations appears to vary between bacterial species and experimental conditions. Mixed-ligand siderophore conjugates, compounds able to enter through several receptors, or combinations targeting different uptake pathways may therefore be useful approaches to limit resistance. Further studies under infection-relevant conditions will be needed to determine how frequently such resistance occurs and whether loss of ferrichrome uptake imposes a clinically relevant fitness disadvantage.

In the longer term, knowledge of siderophore uptake at the strain level may also allow more precise antimicrobial delivery. Different bacterial species, and even individual strains, vary in the repertoire and expression of siderophore transporters. Rapid identification of functional uptake systems in clinical isolates could therefore help to select siderophore carriers that match the transport profile of a particular pathogen. Such an approach remains hypothetical, but indicates a possible route toward a more selective use of siderophore-based antimicrobials.

For diagnostic applications, improving metabolic stability while retaining strong metal binding and appropriate pharmacokinetics will remain a central goal. The contrasting *in vivo* behavior of closely related compounds such as ferrirubin and ferrirhodin illustrates how strongly small structural differences can influence imaging properties. In cancer research, further studies in immunocompetent models will be particularly important to separate direct cytotoxic effects from immune-mediated mechanisms and to determine whether the reported synergy with established therapies can be reproduced across different tumor types.

Practical constraints will ultimately determine which of these approaches reach application. Access to material remains a bottleneck. The clinical development of albomycin was limited primarily by low production yields rather than by insufficient activity, and current synthetic routes to ferrichrome analogs, although far more efficient than the early solution-phase syntheses, still rely on specialized building blocks. Scalable and cost-effective synthesis, together with systematic data on serum stability, plasma protein binding, and host toxicity, will therefore be as decisive as further gains in uptake efficiency. So far, no ferrichrome-based conjugate has entered clinical evaluation, and clinical experience with siderophore-mediated antibacterial delivery is still limited to the catecholate conjugate cefiderocol.

Overall, future progress will depend on bringing together synthetic chemistry, structural biology, microbiology, and computational approaches. A clearer definition of the structural requirements for metal coordination, receptor recognition, and cargo delivery should allow ferrichrome-derived systems to move from largely empirical design toward more rational, application-specific platforms.
